# Rescue strategy for advanced liver malignancy with retrohepatic inferior vena cava thrombi: experience to promote surgical oncological benefit

**DOI:** 10.1186/s12957-017-1145-0

**Published:** 2017-04-12

**Authors:** Meng-Hsing Ho, Teng-Wei Chen, Kuang-Wen Ou, Jyh-Cherng Yu, Chung-Bao Hsieh

**Affiliations:** 1 Division of General Surgery, Department of Surgery, Tri-Service General Hospital, No. 325, Sec. 2, Cheng-Kung Road, Neihu 114, Taipei, Taiwan People’s Republic of China; 2 Divisions of Plastic Surgery and General Surgery, Department of Surgery, Tri-Service General Hospital, National Defense Medical Center, Taipei, Taiwan People’s Republic of China

**Keywords:** Liver malignancy, IVC thrombi, Surgical procedure, Locoregional therapy

## Abstract

**Background:**

The prognosis of advanced liver malignancy with inferior vena cava (IVC) thrombi is poor. Many therapeutic policies are challenging for long-term prognosis. We performed the modified effective technique of transdiaphragmatic intrapericardial IVC isolation for curative resection of IVC tumors and prolonged survival time.

**Methods:**

Between 2003 and 2015, 10 patients, sustained liver malignancy with IVC thrombi, underwent surgical intervention. Liver resection with thrombectomy under total hepatic vascular exclusion via the transdiaphragmatic intrapericardial IVC isolation method was performed for these 10 patients. The first 4 patients underwent retrohepatic IVC resection in order to complete resection, and the other 6 patients preserved the retrohepatic IVC. The last 3 patients received preoperative locoregional therapies, and all 10 patients received postoperative adjuvant chemotherapies immediately.

**Results:**

All 10 patients underwent gross en bloc tumor resections with thrombectomy with R0 resection. There was no surgical mortality. Shortening of operation time and reduction of both intraoperative blood loss and hospital stay were demonstrated in the last 6 patients with preserving the retrohepatic IVC. However, similar time to recurrence and survival time were noted in the first 7 patients. The last 3 patients, who had received preoperative locoregional therapies, have better disease-free survival time.

**Conclusion:**

Simplified surgical procedure combined with preoperative locoregional therapies and rapid postoperative adjuvant treatment may provide a greater advantage for these patients.

**Electronic supplementary material:**

The online version of this article (doi:10.1186/s12957-017-1145-0) contains supplementary material, which is available to authorized users.

## Background

Liver malignancy with inferior vena cava (IVC) thrombi has a poor prognosis [1] and is usually considered terminal. Without curative resection, the long-term prognosis for these patients is dismal. Repeated pulmonary emboli also frequently imperil patients and can cause sudden death. The advances in surgical and anesthetic techniques have made curative surgery feasible for selected patients [2–5]. However, many therapeutic modalities are applied as rescue treatments, such as transarterial chemoembolization (TACE), radiofrequency ablation, or radiotherapy. Adjuvant chemotherapy or target therapy also retards tumor progression. A combination of these therapies seems as the silver lining for patients with advanced liver malignancy if curative surgery is possible.

The difficulties associated with surgical intervention are the invasion of adjacent organs, the various tumor histopathologic types, and the location of thrombus. It is challenging for the surgeon to remove the tumor completely, but this affects the prognosis. Liver resection under total hepatic vascular exclusion (THVE) makes such a complete resection possible [3, 6]. Sternotomy with intra-pericardial IVC isolation has been performed to achieve a safe margin and to prevent an embolus from the thrombus. We have previously introduced the technique of transdiaphragmatic intrapericardial IVC isolation for resection of IVC tumors [7]. This method can successfully achieve the resection of gross tumor en bloc and avoid the need for sternotomy and cardiopulmonary bypass. However, what is critical is how to prolong the survival time or time to recurrence. Here, we present our preliminary experience with managing advanced liver malignancy with IVC thrombus.

## Methods

### Patients

**Fig. 1:**
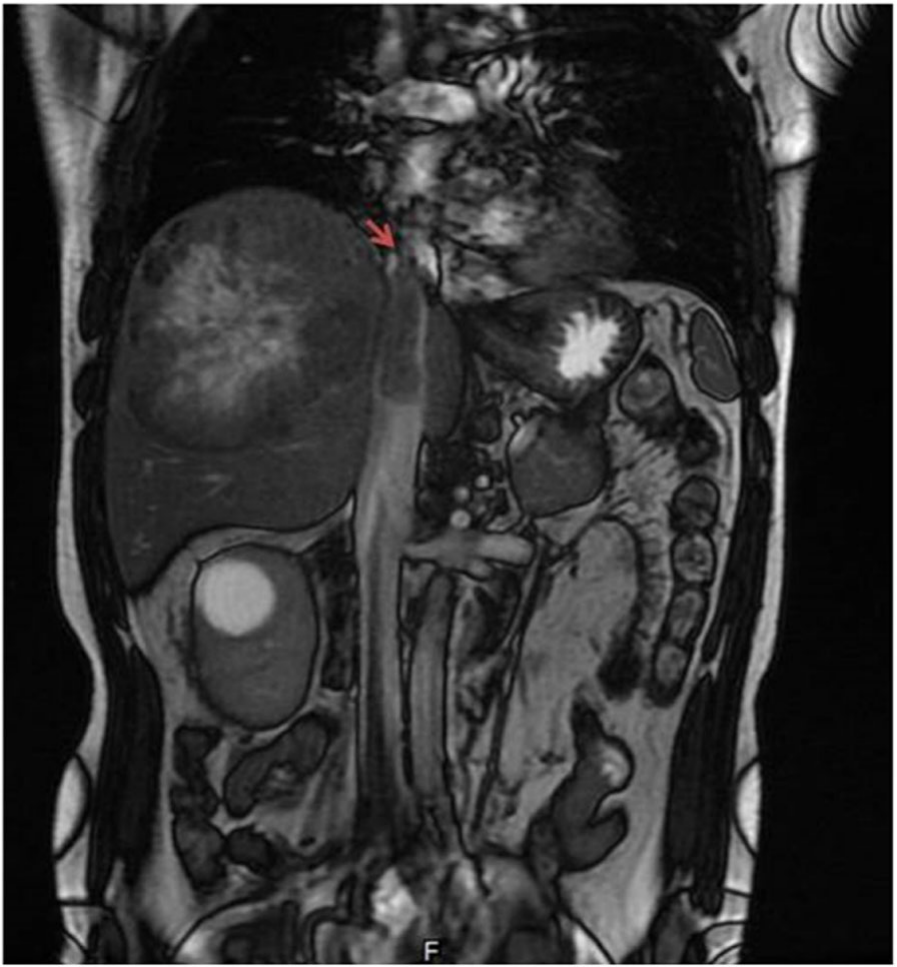
Magnetic resonance imaging of a patient with hepatocellular carcinoma. The *arrow* indicates the thrombi reaching the hepatocaval junction

Between 2003 and 2015, 10 patients were diagnosed with advanced liver malignancy (8 with hepatocellular carcinoma (HCC), 1 with a leiomyosarcoma, and 1 with an adrenocortical carcinoma) and underwent liver resection with thrombectomy under THVE via the transdiaphragmatic intrapericardial IVC isolation method at the Tri-Service General Hospital in Taiwan. All patients had a tumor thrombus within the retrohepatic IVC, extending near or to the hepatocaval junction (Fig. 1). Four of them underwent resection of the retrohepatic IVC and reconstruction in order to complete resection [7]. Demographic data of the patients in this study are presented in Table 1. There were seven men and three women, with a mean age of 52.8 years (SD 17.7 years; range, 29–86 years). Five patients underwent right lobectomy of the liver, 3 patients underwent extended right lobectomy of the liver, and 2 patients underwent extended left lobectomy of the liver. We used the method of transdiaphragmatic intrapericardial IVC isolation to perform THVE and removed the IVC thrombus via the backflow of the inferior phrenic veins and other collateral vessels. The study was approved by the institutional review board I of Tri-Service General Hospital, National Defense Medical Center (TSGHIRB No. 1-104-05-136).

**Table 1:** Demographic data of patients undergoing curative surgery

Patient	Sex	Age (years)	Diagnosis	Child class (score)	Tumor marker	Location of hepatic tumor	Thrombi location	Preoperative therapy
1	F	68	Leiomyosarcoma of IVC with liver invasion	Non-cirrhosis	N/A	Seg 1,2,3	Reaching the hepatocaval junction	N
2	F	46	HBV-related HCC	A [5]	AFP 5000 ng/dL	Seg 7,8	Near the hepatocaval junction	N
3	M	57	HBV-related HCC	A [6]	AFP 4000 ng/dL	Seg 5,6,7,8	Reaching the hepatocaval junction	N
4	F	38	Adrenocortical carcinoma with liver	Non-cirrhosis	N/A	Seg 4,5,6,7,8	Reaching the hepatocaval junction	N
5	M	72	HBV-related HCC	A [5]	AFP >40,000 ng/dL	seg 4,5,6,7,8	Near the hepatocaval junction	N
6	M	46	HBV-related HCC	A [5]	AFP >40,000 ng/dL	seg 7	Near the hepatocaval junction	N
7	M	29	HBV-related HCC	A [5]	AFP >40 000 ng/dL	seg 5,6,7,8	Near the hepatocaval junction	N
8	M	46	HBV-related HCC	A [5]	AFP >40,000 ng/dL	seg 6,7,8	Reaching the hepatocaval junction	TACE + sorafenib
9	M	86	Non-HBV or –HCV-related HCC, sacromatoid type	A [5]	AFP 2.29 ng/dL	seg 7,8	Reaching the hepatocaval junction	TACE + Sorafenib
10	M	40	HBV-related HCC	A [5]	AFP 168.5 ng/dL	seg 5,6	Reaching the hepatocaval junction	TACE + sorafenib
Mean		52.8						
SD		17.7						

*AFP* alpha-fetoprotein, *HBV* hepatitis B virus, *F* female, *HCC* hepatocellular carcinoma, *HCV* hepatitis C virus, *M* male, *TACE* transarterial chemoembolization

### Preoperative evaluation and management

Routine preoperative evaluation included liver function tests and tumor markers. The ICG15 test was used if major hepatectomy was inevitable. All patients were further investigated by tissue biopsy, bone scan, or positron emission tomography in order to rule out distal metastasis. Abdominal ultrasonography, abdominal computed tomography (CT), and/or magnetic resonance imaging were performed to assess the resectability of liver lesions. Neoadjuvant chemotherapy, TACE, or radiotherapy was considered in order to decrease the tumor burden. Sorafenib was administered to recent HCC patients for local control of disease progression. Only the last 3 patients received preoperative locoregional therapies. Patients who underwent these locoregional therapies were arranged follow-up dynamic CT scans 1 month later to determine the effect of therapies. Subsequently, operations were arranged if the disease was stable.

### Surgical technique

After the induction of general anesthesia, the patient was placed in the supine position. A reversed L-shaped incision or bilateral subcostal incision with upward extension was made, and the organs within the abdominal cavity were inspected. Transesophageal echocardiography was performed at the same time to delineate the cranial extent of the thrombus.

**Fig. 2:**
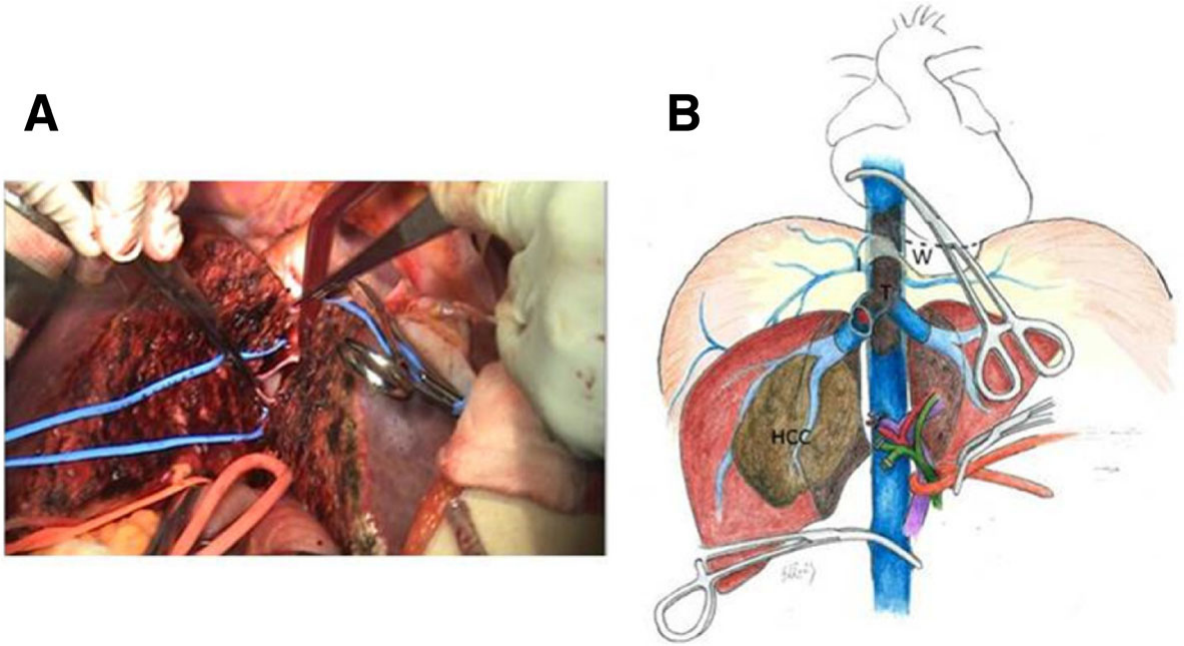
**a** Photograph shows thrombectomy via backflow of the inferior phrenic veins under total hepatic vascular exclusion through a transdiaphragmatic pericardial window. **b** Illustration shows the application of a transdiaphragmatic pericardial window. *I* inferior phrenic veins, *W* transdiaphragmatic pericardial window, *T* IVC thrombi


The plane between the liver and diaphragm was separated carefully. Then, a transdiaphragmatic pericardial window, about 5 × 5 cm in diameter, was created, and the intra-pericardial IVC was looped through the window. The liver dissection line was detected under intraoperative ultrasound guidance. A “tumor non-touch” anterior approach with a liver-hanging hepatectomy was performed with or without the Pringles’ maneuver, and the IVC was exposed. In order to maintain cardiac input after IVC clamping, a rapid blood transfusion pump and inotropic agents need to be prepared thoroughly. No auto-transfusion device was used in order to avoid tumor metastasis. The central venous pressure was maintained at about 5 cm H_2_O, and the mean arterial pressure was maintained at about 60 mmHg before achieving THVE. THVE was achieved by clamping of the portal trial, intra-pericardial IVC, and infra-hepatic IVC. Thrombectomy via backflow of the inferior phrenic veins was done meticulously, and hepatectomy was subsequently carried out (Fig. 2, Additional file 1: Video S1). Some patients needed to undergo segmental resection of the IVC in order to complete tumor resection. After cleaning the surgical field, another vascular clamp was applied under the junction of the hepatic vein and IVC, and then the intra-pericardial and portal trial clamp were removed to allow liver reperfusion and decrease blood loss from the inferior phrenic vein. The THVE was about 10 min. Finally, peritoneal lavage with distilled water was performed.

Additional file 1: The video showed a 46-year-old man, who has the HBV related advanced HCCs ( seg 6,7,8) with retrohepatic IVC thrombi, underwent surgical intervention (Liver resection with thrombectomy under total hepatic vascular exclusion via the transdiaphragmatic intrapericardial IVC isolation method). (MP4 31682 kb)


### Postoperative management and follow-up monitoring

After operation, thalidomide or sorafenib was prescribed for patients with HCC, and adjuvant therapies were also applied for other patients persistently if required by the condition. All patients received adjuvant therapy after surgery, including monitored for tumor recurrence by abdominal ultrasonography and measurement of tumor markers every month or by CT every 3 months. Suspected intrahepatic recurrence was confirmed by hepatic angiography or dynamic CT. Suspected extrahepatic recurrence was confirmed by elevated tumor marker, positron emission tomography, and tumor biopsy.

## Results

In this study, gross en bloc tumor resection with thrombectomy was performed on all 10 patients. Preoperative, intraoperative, postoperative, and follow-up data for the patients are provided in Table 2. The mean operative time was 494.4 min (SD, 201.8 min; range, 302–825 min) and mean blood loss was 1245 mL (SD, 1004.9 mL; range, 500–3900 mL). Perioperative massive bleeding and greater blood transfusion occurred only in the leiomyosarcoma and adrenocortical carcinoma patients. According to Clavien-Dindo classification, three major complications (grade ≥3a) were observed in 3 patients. Only 1 patient underwent reoperation due to delayed bleeding. All patients underwent a R0 resection. The mean length of hospital stay was 14.6 days (SD, 8.1 days; range, 8–30 days). There was no surgery-related mortality. Shortening of the operation time, reduced intraoperative blood loss, and hospital stay were obviously noted in preserved retrohepatic IVC groups.(Table 3) The first 7 patients sustained intrahepatic recurrence and/or distal metastasis several months later and subsequent liver failure, or even death. The last 3 patients are alive and disease-free without events until the present.

**Table 2:** Intraoperative, postoperative, and follow-up data of patients undergoing curative surgery

Patient	Surgery	Operation time (min)	Blood loss (mL)	Complication (CD)	Postoperative tumor marker (1 month)	Postoperative hospital stay (days)	Adjuvant therapy	Disease-free time (months)	Survival time (months)
1	ELL + I + T	825	1850	IIIa	N/A	30	RT	3	6
2	RL + I + T	725	630	I	AFP 175.5 ng/dL	10	Thalidomide	5	9
3	RL + I + T	721	850	IIIb	AFP 188.5 ng/dL	29	Thalidomide	12	15
4	A + ERL + I + T	602	3900	IIIa	N/A	13	CT	15	17
5	ERL + D + T	402	500	I	AFP 218 ng/dL	10	Thalidomide	8	14
6	RL + T	350	1000	Nil	AFP 450 ng/dL	12	Thalidomide	9	17
7	ERL + T	377	900	I	AFP 500 ng/dL	10	Thalidomide	5	15
8	RL + T	302	1200	Nil	AFP 12.7 ng/dL	8	Sorafenib	39+	Alive
9	ELL + T	328	920	II	AFP <0.5 ng/dL	15	Sorafenib	30+	Alive
10	RL + T	312	700	Nil	AFP 5.27 ng/dL	9	Sorafenib	10+	Alive
Mean		494.4	1245			14.6			
SD		201.8	1004.9			8.1			

*ELL* extended left lobectomy, *I* IVC resection, *T* thrombectomy, *A* adrenectomy, *D* partial diaphragm resection, *RL* right lobectomy, *ERL* extended right lobectomy, *CD* Clavien-Dindo classification, *N/A* no analysis, *RT* radiotherapy, *CT* chemotherapy, *+* the duration to date of article submission

**Table 3:** Subgroups of patients undergoing curative surgery and analysis of intraoperative, postoperative, and follow-up data

	IVC resection *N* = 4	Preserved IVC *N* = 3	Preserved IVC+ preoperative palliative treatment *N* = 3
Operation time (min) Mean (SD)	718.25 (91.22)	376.33 (26.01)	314 (13.11)
Blood loss (mL) Mean (SD)	1807.5 (1492.61)	800 (264.58)	940 (250.60)
Postoperative tumor marker (1 month) Mean (SD)	N/A	389.33 (150.47)	6.16 (6.15)
Postoperative hospital stay (days) Mean (SD)	20.5 (10.47)	10.67 (1.15)	10.67 (3.79)
Disease-free time (months) Mean (SD)	8.75 (5.68)	7.33 (2.08)	26.33 (14.84) at least
Survival time (months) Mean (SD)	11.75 (5.12)	15.33 (1.52)	26.33 (14.84) at least

## Discussion

The long-term prognosis for advanced liver malignancy, no matter primary or secondary lesions, with IVC thrombus is poor. Patients often manifest pain, IVC obstruction syndrome, and repeating pulmonary emboli. Surgical resection of these tumors is not recommended as a standard treatment, but it should be considered for relieving the symptoms if all other treatment options have been exhausted. However, there was no significant long-term surgical oncological benefit proved in the past study, even if R0 resection was achieved. In our preliminary experience, the combined therapies, including preoperative locoregional therapy, surgical resection with a free margin, and adjuvant therapies as soon as possible postoperatively, might be the only life-saving therapy.

Most of the patients in our center received palliative locoregional treatments [8], such as radiotherapy, TACE, or targeted therapy, and expired abruptly. In contrast, a few patients had good responses to the locoregional therapies. These differences may be attributed to a “selection effect.” During palliative treatments, some patients might become despaired due to tumor progression, and thus selecting patients with slower tumor progression, and therefore relatively benign tumor behavior. In addition, the locoregional therapies have been reported to reduce the tumor burden and improve survival rate for HCC patients who are undergoing liver transplant. [9–11] It also makes the operation more smoothly. Subsequently, surgical resection might be considered. After meticulous assessment, attempted curative resection might be performed. In the past, these patients might have undergone such procedures as hepatectomy with or without THVE, IVC resection, sternotomy, or thoracotomy with cardiopulmonary bypass. However, possible surgical complications, residual tumor retention, complicated operation procedures, and long recovery time might cause these patients to be ineligible for the next treatment and to become hopeless.

**Fig. 3:**
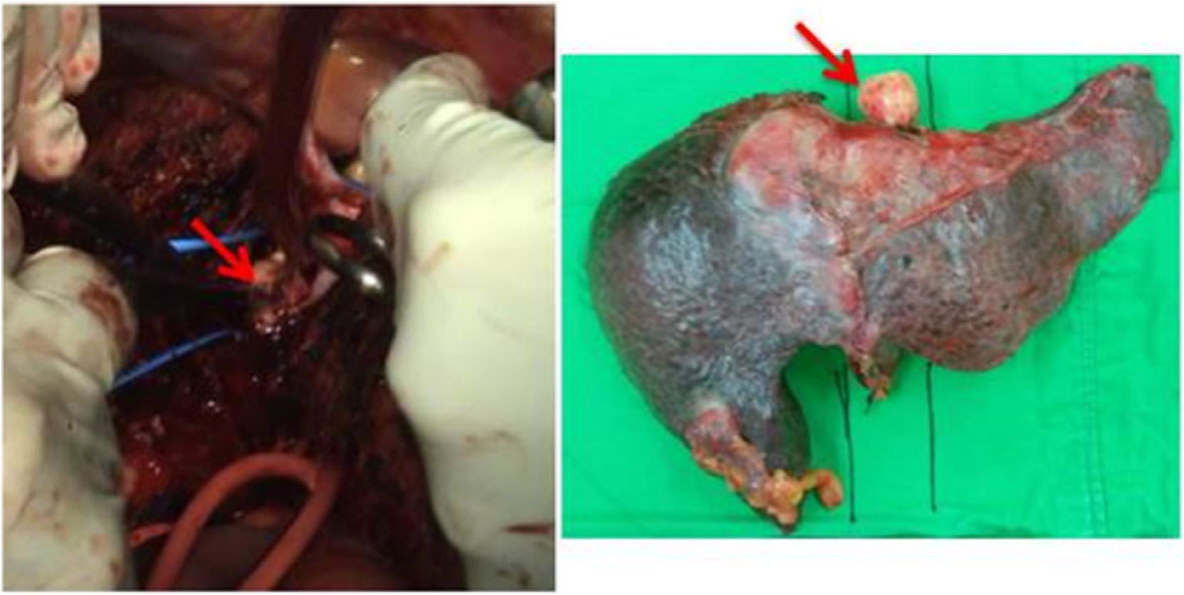
The *arrow* indicates the thrombus, which seemed solid and non-adherent

In our study, liver malignancies with IVC thrombosis were found in the diagnosis of all patients and even extended to the hepatocaval junction. We performed liver resection with thrombectomy under THVE by intrapericardial IVC isolation through a transdiaphragmatic pericardial window. We sought to avoid possible complications related to sternotomy or thoracotomy and diminish patients’ fear. At first, we also resected the retrohepatic IVC in order to completely remove the tumor [7]. However, this caused the surgical procedure to be more difficult. Subsequent anticoagulation prescription and possible graft thrombosis also affected patients with postoperative dysfunctional liver. Therefore, we preserved the retrohepatic IVC for the patients and performed thrombectomy via backflow of the inferior phrenic veins and other collateral vessels. These patients seemed to recover more rapidly and to have less worry about surgical procedure-related complications. Initially, the survival time or disease-free time was similar between groups. However, the last 3 patients all received preoperative palliative treatments for reducing tumor burden and relieving symptoms. After observation for 1 month, they underwent surgical intervention if no obvious tumor progression or distal metastasis developed. We noted that the intraoperative blood loss was decreasing and the thrombus seemed solid and non-adherent (Fig. 3). These changes might have been caused by locoregional treatment-related ischemic changes or lipiodol retention. The preoperative locoregional therapies reduced the tumor burden and made the thrombi non-adherent. Attempting complete thrombectomy and detachment of the space between the IVC and tumor became easier. It also effectively shortened the operation time, and no major complication developed. The operation process became more simplified and effective. There is also a trend for prolonged time to recurrence in the last 3 patients.

Our surgical technique is a simplified and effective procedure with an acceptable operation time and amount of blood loss. There was no procedure-related mortality. Most of these patients received adjuvant treatments about 2 weeks postoperatively. The operation time and hospital stay were shortened considerably. Tumor makers decreased dramatically, and disease-free time or survival time improved, especially in the last 3 patients.

**Fig. 4:**
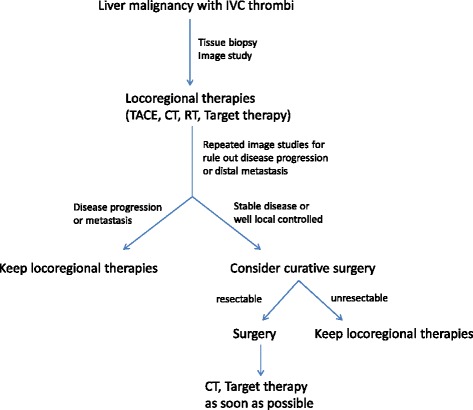
Flowchart applied in our center for liver malignancy with IVC thrombi. *TACE* transarterial chemoembolization, *CT* chemotherapy, *RT* radiotherapy

On the basis of various tumor histopathologic types, the prognosis varies slightly. For HCC with IVC thrombi, the survival span is less than 3 months without surgical intervention. For leiomyosarcoma with IVC thrombi, the medial survival is only 1 month without operation. Therefore, survival is usually measured in months in untreated liver malignancy. Palliative treatments are applied to retard disease progression and provide a selection effect. However, many small series have demonstrated a surgical benefit for these patients if the difficulty of the surgical procedure can be overcome [2, 4, 12–16]. In their HCC group, Kokudo et al. [12] demonstrated that IVC thrombosis and R1/2 resection were significant risk factors for recurrence and survival. They also revealed that the mean survival time and time to recurrence in HCC patients with IVC thrombi were 1.39 and 0.25 years respectively. Then, prolonging survival time became the critical issue. From our preliminary experience, a simple and effective surgical procedure combined with preoperative locoregional therapies and rapid postoperative adjuvant treatment may provide more advantages to these patients (Fig. 4). However, this is a small series retrospective study, caused some limitation. Further larger study is needed to clarify the benefit of this therapeutic policy for advanced liver malignancy with retrohepatic IVC thrombi.

## Conclusion

Curative surgical intervention for liver malignancy with IVC thrombus resulted in a survival benefit. The issue of prolonged survival time remains a challenge for physicians. We believe that a simple and effective surgical procedure combined with preoperative locoregional therapies and rapid postoperative adjuvant treatment may provide greater advantage to such patients.

## Abbreviations

CT: Computed tomography
HCC: Hepatocellular carcinoma
IVC: Inferior vena cava
TACE: Transarterial chemoembolization
THVE: Total hepatic vascular exclusion
