# Erratum to: Rescue strategy for advanced liver malignancy with retrohepatic inferior vena cava thrombi: experience to promote surgical oncological benefit

**DOI:** 10.1186/s12957-017-1156-x

**Published:** 2017-04-28

**Authors:** Meng-Hsing Ho, Teng-Wei Chen, Kuang-Wen Ou, Jyh-Cherng Yu, Chung-Bao Hsieh

**Affiliations:** 1 Division of General Surgery, Department of Surgery, Tri-Service General Hospital, National Defense Medical Center, Taipei, Taiwan, Republic of China; 2 Divisions of Plastic Surgery and General Surgery, Department of Surgery, Tri-Service General Hospital, National Defense Medical Center, Taipei, Taiwan, Republic of China

## Erratum

Upon publication of the original article [1], an error was discovered in Table 1 (the tumor location of the 9th patient). This mistake was owing to typing error. This has now been corrected in this erratum and the original manuscript has been updated. We apologise for any inconvenience caused by this error.

**Table 1:** Demographic data of patients undergoing curative surgery

Patient	Sex	Age (y)	Diagnosis	Child class (score)	Tumor marker	Location of hepatic tumor	Thrombi location	Pre-operative therapy
1	F	68	Leiomyosarcoma of IVC with liver invasion	Non-cirrhosis	N/A	Seg 1,2,3	Reaching the hepatocaval junction	N
2	F	46	HBV-related HCC	A (5)	AFP: 5000 ng/dL	Seg 7,8	Near the hepatocaval junction	N
3	M	57	HBV-related HCC	A (6)	AFP: 4000 ng/dL	seg 5,6,7,8	Reaching the hepatocaval junction	N
4	F	38	Adrenocortical carcinoma with liver	Non-cirrhosis	N/A	seg 4,5,6,7,8	Reaching the hepatocaval junction	N
5	M	72	HBV-related HCC	A (5)	AFP: >40 000 ng/dL	seg 4,5,6,7,8	Near the hepatocaval junction	N
6	M	46	HBV-related HCC	A (5)	AFP: >40 000 ng/dL	seg 7	Near the hepatocaval junction	N
7	M	29	HBV-related HCC	A (5)	AFP: >40 000 ng/dL	seg 5,6,7,8	Near the hepatocaval junction	N
8	M	46	HBV-related HCC	A (5)	AFP: >40 000 ng/dL	seg 6,7,8	Reaching the hepatocaval junction	TACE + Sorafenib
9	M	86	Non-HBV or –HCV-related HCC, sacromatoid type	A (5)	AFP: 2.29 ng/dL	seg 4	Reaching the hepatocaval junction	TACE + Sorafenib
10	M	40	HBV-related HCC	A (5)	AFP: 168.5 ng/dL	seg 5,6	Reaching the hepatocaval junction	TACE + Sorafenib
Mean		52.8						
SD		17.7						

*AFP* alpha-fetoprotein, *HBV* hepatitis B virus, *F* female, *HCC* hepatocellular carcinoma, *HCV* hepatitis C virus, *M* male, *TACE* transarterial chemoembolization
